# Mathematical Model of SARS-Cov-2 Propagation Versus ACE2 Fits COVID-19 Lethality Across Age and Sex and Predicts That of SARS

**DOI:** 10.3389/fmolb.2021.706122

**Published:** 2021-07-12

**Authors:** Ugo Bastolla

**Affiliations:** Centro de Biologia Molecular “Severo Ochoa”, CSIC-UAM Cantoblanco, Madrid, Spain

**Keywords:** SARS-CoV-2, ACE2, viral propagation, mathematical model, inflammatory response, COVID-19

## Abstract

The fatality rate of Covid-19 escalates with age and is larger in men than women. I show that these variations correlate strongly with the level of the viral receptor protein ACE2 in rat lungs, which is consistent with the still limited data on human ACE2. Surprisingly, lower receptor levels correlate with higher fatality. I propose two possible explanations of this negative correlation: First, a previous mathematical model predicts that the velocity of viral progression in the organism as a function of the receptor level has a maximum and declines for abundant receptor. Secondly, degradation of ACE2 by the virus may cause the runaway inflammatory response that characterizes severe CoViD-19. I present here a mathematical model that predicts the lethality as a function of ACE2 protein level based on the two above hypothesis. The model fits Covid-19 fatality rate across age and sex in three countries with high accuracy ($r^{2}>0.9$) under the hypothesis that the speed of viral progression in the infected organism is a decreasing function of the ACE2 level. Moreover, rescaling the fitted parameters by the ratio of the binding rates of the spike proteins of SARS-CoV and SARS-CoV-2 allows predicting the fatality rate of SARS-CoV across age and sex, thus linking the molecular and epidemiological levels.

## 1 Introduction

The Covid-19 pandemics (Zhou et al., 2020) has caused millions fatalities worldwide (Dong et al., 2020), creating a tremendous threat to global health. It presents a strong gradient of fatalities across age and a sex bias with much higher severity in males than females. Analysis of seroprevalence studies (Pastor-Barriuso et al., 2020) and modelling studies that extrapolate the number of infections (Davies et al., 2020) indicate that, at young age, most SARS-CoV-2 infections are asymptomatic and the fatality rate is very low, whereas for the elderlies most infections are severe and a large fraction of them can be fatal. Understanding the biological reasons that underlie these striking differences is one of the most pressing problems of CoViD-19 research, which might lead to better prediction of the disease prognosis and possible treatments that approach the severity of the worst affected groups to that of the most protected ones.

Here I show that the case fatality rate of Covid-19 across age and sex correlates negatively with the level of the protein Angiotensin converting enzyme 2 (ACE2), the cellular receptor both of SARS and SARS-CoV-2 virus (Hamming et al., 2004; Zhou et al., 2020), which belongs to the anti-inflammatory axis of the Renin-Angiotensin-System (RAS) (Paz Ocaranza et al., 2020). The correlation is very strong with membrane-bound ACE2 protein in rat lungs, which decreases with age and is higher in old females than old males (Xie et al., 2006). The same qualitative pattern is observed for membrane-bound ACE2 in mice (Yoon et al., 2016), where all the anti-inflammatory axis of the RAS decreases with age. Data on ACE2 expression through age in humans were not available until recently, but the Covid-19 pandemics brought an explosion of studies. Despite apparently contradictory conclusions, all studies are consistent with a model in which ACE2 expression starts in late foetal life (Muus et al., 2020), it is lower in young children than in adults (Bunyavanich et al., 2020; Saheb Sharif-Askari et al., 2020), it reaches a maximum at young age and then it decreases during adulthood age both at the level of mRNA (Chen et al., 2020) and at the level of membrane-bound protein (Zhang et al., 2021). Thus these data support similar qualitative trajectories of ACE2 expression in rodents and humans, as hypothesized here and further discussed later. ACE2 is removed from the cell membrane by the metalloprotease ADAM17 (Lambert et al., 2005; Xu et al., 2017) whose expression increases with age (Dou et al., 2017; Liu et al., 2019), thereby suggesting that the rate of degradation increases with age.

The negative correlation between ACE2 and lethality is surprising: higher levels of the receptor decrease the lethality exponentially. The apparent paradox can be reconciled through a mathematical model of viral infection, developed before the COVID-19 pandemics, which predicted how viral propagation in the organism depends on the adsorption rate of viruses on cells (Fort and Méndez, 2002). Here I express this model in terms of receptor level and show that it predicts that the speed of viral propagation is a non-monotonic function of the receptor level, which reaches a maximum and decreases for high receptor expression.

The second mechanism that may underlie the negative correlation concerns the function of ACE2 not as viral receptor but as key enzyme for controlling the pro-inflammatory peptides of the RAS and the bradykinin system. Besides regulating blood pressure (BP) and electrolyte homeostasis in blood, the RAS (Paz Ocaranza et al., 2020) plays a central role in inflammatory processes (Agarwal et al., 2013), immune response (Satou et al., 2018) and coagulation (Lip, 2000; Remková and Remko, 2010), which characterize the most severe Covid-19 cases (Diamond, 2020; Ingraham et al., 2020). Its main player is the family of peptides derived from angiotensin I (Ang1-10), cleaved by the enzyme Renin from the protein angiotensinogen. Its pro-inflammatory arm is constituted by angiotensin II (Ang1-8), cleaved from Ang1-10 by the angiotensin converting enzyme (ACE) homologous to ACE2. Ang1-8 bound to the receptor ATR-1 triggers a cascade of reactions leading not only to vasoconstriction and increased BP but also to activation of the transcription factor NFkB that upregulates inflammatory cytokines (IL-1, TNF-α and IFN-γ among others), activates white blood cells and platelets, and favours thrombotic processes (Brasier, 2010). The enzyme ACE2 limits the level of Ang1-8 by converting its precursor Ang1-10 to Ang1-9 (Donoghue et al., 2020) that is subsequently cleaved by ACE to Ang1-7, and by directly converting Ang1-8 to Ang1-7, which belongs to the anti-inflammatory arm of the RAS since it favours vasodilation, reduces BP and attenuates inflammation (Chappell and Al Zayadneh, 2017).

**FIGURE 1:**
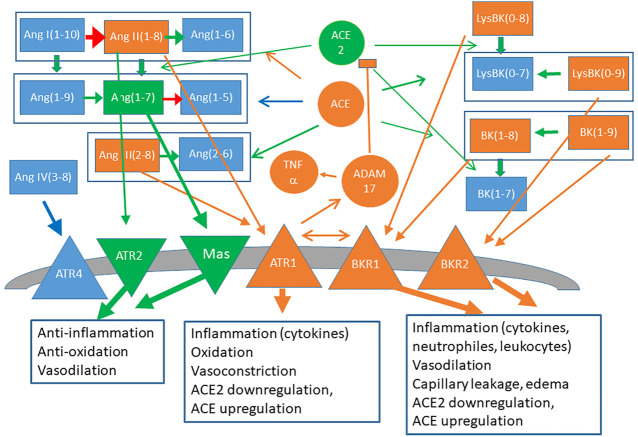
Schematic representation of the RAS **(left)** and bradykinin **(right)** systems. Signaling peptides are represented as rectangles, peptidases (ACE, ACE2), proteases (ADAM17) and cytokines (TNFα) are represented as circles and membrane receptors are represented as triangles. Components and links with mainly proinflammatory character are depicted in orange and anti-inflammatory components are depicted in green.

The bradykinin system has vasodilatory effects that lower blood pressure and it is very strongly coupled with the RAS. It consists of two axis. The first axis is mediated by the pro-inflammatory peptide des-Arg^9^-bradykinin (DABK), which is downregulated by ACE2 and whose receptor BK1R is upregulated upon inflammation by Ang1-8 (in turn downregulated by ACE2) bound to the receptor ATR1. Stimulation of this axis leads to release of pro-inflammatory chemokines, lung inflammation and injury (Sodhi et al., 2018). The second axis is mediated by the peptide bradykinin (BK), which is downregulated by ACE and whose receptor BK2R is in turn activated by Ang1-7 and Ang1-9 produced by ACE2 and is stimulated by Ang1-8 bound to the receptor ATR2 (Kurisu et al., 2003). Therefore, ACE2 channels bradykinin from the first axis to the second one. A summary of the main inflammatory and anti-inflammatory components of the angiotensin and bradykinin system is represented in Figure 1.

Upon viral entry the spike protein of SARS-CoV and probably also SARS-CoV-2 cause the internalization and degradation of ACE2 (Kuba et al., 2005) that critically contributes to lung damage (Imai et al., 2005; Imai et al., 2008; Jia, 2016). Decrease of ACE2 raises the severity of lung injury in other inflammatory diseases (Jia, 2016) and in aging rats (Schouten et al., 2016), which may be explained by the increase of Ang1-8 and its adverse effects.

Here I develop a set of mathematical models of SARS-CoV-2 lethality versus the pre-infection level of ACE2 based on two aspects: the influence of ACE2 on viral progression (Fort and Méndez, 2002) and the negative effect of its degradation. These models are fitted to the CFR of SARS-CoV-2 across six classes of age and sex in Italy, Spain and Germany, and support the hypothesis that the receptor level slows down the virus propagation in the infected organism, which fits the data better than the competing hypothesis that the viral progression is independent of the receptor level and ACE2 influences the lethality only through its negative effect on the inflammatory process. Furthermore, under the same hypothesis and by rescaling the parameters fitted to SARS-CoV-2 by the ratio between the binding rates of the spike proteins of SARS-CoV and SARS-CoV-2, the model predicts well the CFR of SARS-CoV, supporting again the negative relationship between receptor level and virus propagation.

## 2 Results

### 2.1 CoViD-19 Lethality Correlates Negatively With ACE2 Level

The level of the ACE2 protein in rat lungs were quantified across three adult age classes of the two sexes by Xie et al. (2006), who found that it strongly decays with age and it is higher in female than male rats, with largest difference in the oldest cohort where the expression is almost double for females. A similar pattern was observed in mice (Yoon et al., 2016). Observations on ACE2 protein in human lungs (Zhang et al., 2021) and ACE2 mRNA in the GTEx database (Chen et al., 2020) suggest that human ACE2 levels across age and sex are qualitatively similar to rodent data, apart for multiplicative factors that may depend on the organ: they decay with age and they are higher in females than in males. In children the situation is more complicated, since ACE2 protein in serum (Pavel et al., 2021) and ACE2 mRNA (Bunyavanich et al., 2020; Muus et al., 2020; Saheb Sharif-Askari et al., 2020) is lower in children than adults, indicating a non-monotonic trend with age. In fact, it has been observed that ACE2 starts being expressed in late foetal stage and reaches a cell-type dependent maximum at young age (Muus et al., 2020). ACE2 is shed from cell membranes to the serum through the protease ADAM17 (Xu et al., 2017), whose expression increases with age (Dou et al., 2017; Liu et al., 2019). As discussed later, this fact leads to predict that ACE2 levels in cell membranes attain their maximum at younger age than ACE2 mRNA and their decrease is faster. However, in comparison with adults, young children present lower ACE2 levels and reduced Covid-19 severity. We argue later that this reduced severity might be related with the higher expression in children of the alternative angiotensin receptor ATR2 that counteracts inflammation (Kaschina et al., 2017), which can be expected to alleviate the inflammatory consequences of low levels of ACE2.

**FIGURE 2:**
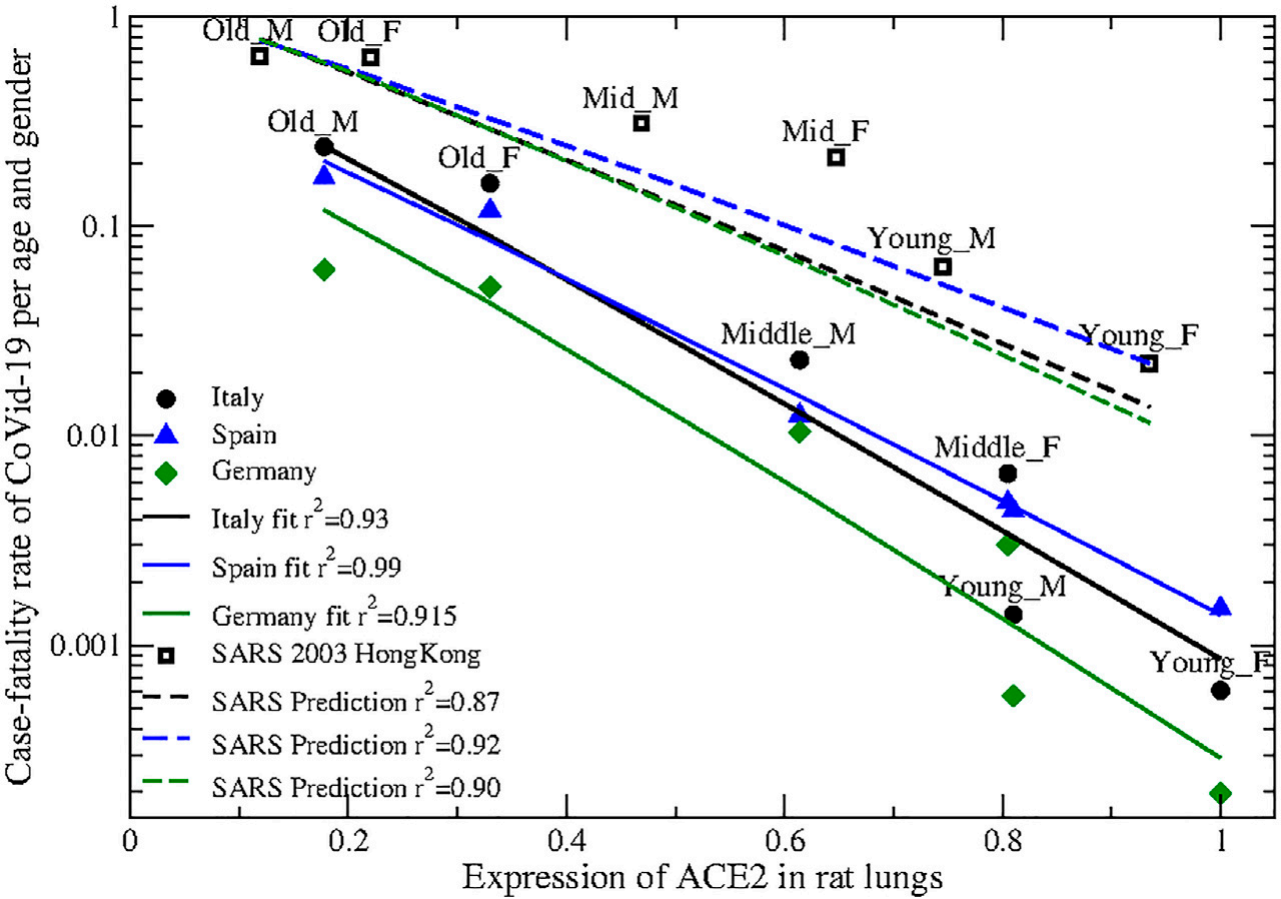
Expression of the ACE2 protein in rats lung (horizontal axis), normalized so that the highest expression is one, vs. case fatality rate (vertical axis) of SARS-CoV-2 (Circles: Italy; triangles: Spain; diamonds: Germany) and SARS 2003 (open squares) in three age classes (young 0–29, middle-age 30–59 and old >59) and two sexes. The solid lines represent best fits to the mathematical model CFR$=\text{exp}\left(-a\text{ACE}2+b\sqrt{\text{ACE}2}-c\right)$ (see text) with fitting parameters $a=7.6\pm 0.5,b=1.1\pm 0.4,c=0.52,r^{2}=0.93$ (Italy), $a=6.9\pm 0.5,b=1.1\pm 0.5,c=0.85,r^{2}=0.99$ (Spain) and $a=8.7\pm 1,b=2.0\pm 1,c=1.4,r^{2}=0.91$ (Germany). The parameter *c* was not fitted but determined so that the relative error on the parameters *a* and *b* equals 50%. The dashed lines represent predictions with the parameters *a* and *b* fitted to SARS-CoV-2 rescaled with the ratio between the binding rate constants of SARS and SARS-CoV-2 (see text) and parameter *c* fitted to the 2003 SARS CFR in Hong-Kong, yielding $a=5.7,b=0.93,c=-0.01,r^{2}=0.87$ (from Italy’s fit), $a=5.1,b=0.97,c=-0.10,r^{2}=0.92$ (from Spain’s fit) and $a=6.5,b=1.7,c=-0.58,r^{2}=0.90$ (from Germany’s fit).

Strikingly, the profile of ACE2 in adult rats in Ref. (Xie et al., 2006). is very strongly anti-correlated with the lethality of SARS-CoV-2. Figure 2 represents the level of the ACE2 protein in rats lung [horizontal axis, data from Figure 2 of (Xie et al., 2006)] versus the case fatality rate (CFR) of CoVid-19 registered in Italy (Istituto Superiore di Sanità, 2020), Spain (OPS, 2020) and Germany (Robert Koch Institute, 2020) in three uniform age classes (young, <30, middle-age 30−59 and old >60; vertical axis) of each sex. Data strongly support the exponential decrease of mortality with ACE2 level, with $r^{2}=0.91$, 0.97 and 0.89, respectively, suggesting that variations of ACE2 describe the largest part of the variation of the CFR.

As for other two-parameter fits tested in this work, the fitted exponents for Italy and Germany coincide within the error and the CFR differ only by a multiplicative factor, supporting the robustness of the data. Data from Spain present higher mortality in the young ages, which might be attributed to more frequent undetected cases in young age with lower severity.

### 2.2 Mathematical Model of Covid-19 Lethality

Mathematical models of viral growth consider three processes: virus adsorption into susceptible cells, production of virus by infected cells after a delay time τ, and viral clearance by the immune system (Smith and Perelson, 2011). The simple mean-field model that does not consider explicit space predicts a minimal receptor density below which the virus does not grow and above which higher receptor levels accelerate the viral progression.

Considering spatial diffusion modifies this situation. Fort and Méndez proposed in 2002 a model of viral propagation through spatial diffusion on susceptible cells and showed that its solution can be expressed as a wave of reproducing viral particles that propagates in space with wave velocity *v* (Fort and Méndez, 2002), for which they were able to find an analytical approximation in terms of the parameters of the model: The adsorption rate $k_{1}$, the rate $k_{2}$ of virus production by infected cells, the initial concentration of susceptible cells $U_{0}$, the viral yield *Y*, i.e., the average number of infective viral particles produced by an infected cell, and the delay time τ between viral entry and release of viral particles. They tested this solution with experiments on the spread of bacteriophages on lysis plaques (Ortega-Cejas et al., 2004). Although developed for bacteriophages, this mathematical model is conceptually applicable to the present setting, except that it considers a one-dimensional reaction diffusion system whose extrapolation to fractal structures such as the lungs is an approximation that may be not accurate (Ben Avraham and Havlin, 2005). Nevertheless, since the functional form that I derived from the model and fitted to the data does not depend on the value of the fractal dimension, I expect that the qualitative results presented here are not influenced by this approximation but only depend on the qualitative dependence between the viral progression in the organism and the receptor level.

Here I express the mathematical solution to the wave velocity in terms of receptor density, assuming that the adsorption rate $k_{1}$ is proportional to the receptor level *A* expressed in susceptible cells times the association rate between the virus and the receptor, $k_{1}=kA$. The main assumption behind this expression is that the rate limiting step for viral entry in the cell is the binding of the virus to the receptor, since the necessary step of cleavage of the spike protein can be facilitated by several cellular proteases. Even if this assumption is not accurate, I expect that the adsorption rate must be an increasing function of the binding of the virus to the ACE2 receptor.

Varying the receptor level *A* (adsorption rate in the original paper), three regimes appear: 1) When *A* is small the viral velocity *v* increases with *A* less than linearly. 2) For intermediate $kA>1/U_{0}\tau Y$ but small with respect to the rate of virus production, $kA<k_{2}/U_{0}$, the viral velocity reaches a plateau where it is almost independent of *A*. 3) Although not explicitly discussed in Ref. (Ortega-Cejas et al., 2004), the formulas presented there remain valid in the regime where kA is larger than the rate of virus production. In this regime the viral progression slows down with receptor density as $v\propto 1/\sqrt{kA}$ (see Section 4 for the derivation).

The latter result is surprising: how can the virus progress more slowly for increasing receptor level? Since this is a mathematical model, the answer is readily found: in the model, viral particles are consumed when they enter a cell (this is embodied in the term $-k_{1}VU$ in the derivative of the viral concentration *V*) but the viral yield does not increase when a cell is infected multiple times. This assumption is justified if the viral population saturates the synthetic machinery of the cell, ribosomes and RNA polymerases, so that the number of produced viral particle *Y* is the same in case of multiple viral entries. In fact, it was proposed that multiple viral entries in the same cell interfere with viral replication. Several viruses such as HIV (Bour et al., 1995; Michel et al., 2005), measles (Schneider-Schaulies et al., 1995), influenza (Marschall et al., 1997) and hepatitis B (Breiner et al., 2001) downregulate their own receptor, preventing multiple entries. The mathematical result that, after the infection is established, very fast adsorption does not favour the virus, agrees with a recent study that demonstrated the protective effect of high adsorption rate through analytic computation, simulation and experiment (Eriksen et al., 2018).

Next, I consider two models of Covid-19 death. The first model only considers variations of viral propagation with ACE2. Death occurs when the virus propagates through the upper respiratory tract or through endothelial cells, reaches the lungs and infects and destroys a critical fraction *X* of it. In the first model *X* is the same for all patients. The second model considers that ACE2 plays an essential role for reverting the inflammatory process propagated by the peptides of the angiotensin and bradykinin systems that ACE2 downregulates. I hypothesize that, if the ACE2 density in an infected organ is below a critical level $A_{c}$, which for simplicity is assumed to be the same for all patients, the organism cannot stop the inflammatory process triggered by the infection, as suggested by the results of experiments with ACE2 knock-out mice (Imai et al., 2005). Being *A* the initial level of ACE2 and *X* the fraction of ACE2 destroyed by the virus, the density of ACE2 at time *t* is A(1−X(t)). I hypothesize that the inflammatory process cannot be reverted if this quantity drops below $A_{c}$ or, equivalently, if $X>1-A_{c}/A$, i.e., in the second model the critical fraction *X* increases with the pre-infection ACE2 level, making the organism more tolerant to severe Covid-19.

Combining these two assumptions with the three regimes of viral propagation described above gives six mathematical models. For each of them, I compute the time $t_{d}$ after which the virus causes death versus the ACE2 level *A* (see Section 4). If the viral velocity increases with ACE2 $t_{d}$ is a decreasing function of *A*. This behaviour contradicts the data and I shall not consider it further. In the regime in which the viral velocity is independent of *A* $t_{d}$ is an increasing function of *A* only for the model that considers the protective effect of ACE2, which is called Model 1. Model 2 assumes that viral propagation decreases with *A* but neglects the protective effect of ACE2 and Model 3 considers both the decrease of viral propagation and the protective effect of ACE2.

I compute lethality as the probability that $t_{d}$ is smaller than the time $t_{i}$ needed by the immune system for clearing the virus, which I model as a random variable with two possible distributions: 1) The exponential distribution, which is the distribution with maximum entropy for given average value. 2) The Gaussian distribution, which is the simplest distribution that is not peaked at zero value of the variable (in this case, the immune system time $t_{i}$). I adopt the same immune system parameters for all age and sex classes in order to limit the number of free parameters and to test if variations of the receptor level are sufficient to fit the lethality.

The three models, combined with the exponential and the Gaussian distribution, yield six different functional forms of the CFR versus *A* that I fit to the data. Some fits have many free parameters, but simple approximations reduce their number to two (for the exponential distribution) or three (for the Gaussian distribution). Moreover, all fits are strongly regularized with rescaled ridge regression (Bastolla and Dehouck, 2019) to limit overfitting as much as possible (see Section 4).

Under both distributions, and for the CFR of the three countries, model 2 gives better fit than model 1 with the same number of free parameters. Under the exponential distribution (two free parameters), the relative quadratic error of the logarithm for models 1 and 2 is 0.37, 0.07, for Spain, 0.41, 0.14 for Italy and 0.53, 0.17 for Germany, respectively. Adopting the Gaussian distribution requires three parameters, with high risk of overfitting, but it maintains the same ranking: 0.04, 0.01 for Spain, 0.10, 0.04 for Italy and 0.14, 0.06 for Germany, see Supplementary Table S1. Model 3 does not improve the fit above Model 2 despite having one more parameter, which is due to the strong regularization that we apply, and combined with the Gaussian model it yields unrealistic negative parameters that suggest that it has too many parameters for the limited available observations. Model 2G with the Gaussian distribution is $-\text{log}\left(\text{CFR}\right)=aA-b\sqrt{A}+c$, with three parameters. To reduce the number of fitting parameters, I fitted the parameters *a* and *b* for different values of *c*, which was not fitted, and chose the value of *c* that yielded a predefined statistical error of the fitted parameters *a* and *b* instead of minimizing the quadratic error of the fit (see Section 4). This gives slightly better fits than the simple exponential function ($r^{2}>0.92$ for all three datasets, see Supplementary Table S1) and limits the risks of overfitting. These fits are represented by the solid lines in Figure 2. The parameters *a* and *b* fitted from different countries differ only slightly more than their statistical error, which may reflect the different incidence of undetected cases across age classes.

In conclusion, for both distributions the hypothesis that the viral progression decreases with the receptor level (Model 2) fits the data much better than the competing hypothesis that the propagation is independent of the receptor level.

### 2.3 SARS 2003

An important prediction of Models 2 and 3 is that the fit parameters *a* and *b* depend on the adsorption constant per unit receptor *k*, with the fit parameters expressed as a∝k and $b\propto \sqrt{k}$ in Model 2G. Crucially, this prediction can be tested on the CFR of the 2003 SARS coronavirus (Karlberg et al., 2004), which also uses ACE2 as cellular receptor. Under the hypothesis that the rate-limiting step for adsorption is the binding of the spike protein to ACE2, justified if the complex is stable enough to allow membrane fusion, and supported by infection assays, *k* is proportional to the spike binding rate constant, which has been measured with biochemical experiments for both SARS and SARS-CoV-2. This allows rescaling the fit parameters *a* and *b* obtained for SARS-CoV-2 to predict the corresponding parameters for SARS (see Section 4). Multiplying the predicted CFR times a global factor that accounts for undetected cases as the only free parameter, we predict the CFR of SARS with very good accuracy. The relative quadratic error is equal to 0.13, 0.08 and 0.01 using the fit parameters from Italy, Spain and Germany, respectively, see Figure 2, Supplementary Table S1 and Discussion. This result further supports the hypothesis that the viral velocity may be slowed down by increasing receptor level.

### 2.4 NL63

It is natural to extend this analysis to the other human coronavirus that uses ACE2 as a receptor, NL63 that causes common cold and is not generally associated with pneumonia. Its spike protein contains a very stable receptor binding domain of 120 residues that showed high binding affinity for ACE2 (Wu et al., 2009). However, the complete S1 domain of the spike (717 residues) is much less stable and its affinity for ACE2 is so small that it could not be measured with binding assays (Hofmann et al., 2005; Mathewson et al., 2008; Glowacka et al., 2010), and it was conjectured that it is 10–100-fold smaller than that of SARS-CoV (Mathewson et al., 2008). Since the CFR of SARS peaks for old females, whose normalized ACE2 is equal to 22% of the maximum value, the model predicts that this is the level at which SARS-CoV propagates fastest. If the binding affinity of the NL63 spike is at least ten times smaller, the ACE2 level at which NL63 propagates fastest is more than double the highest ACE2 level, implying that NL63 is in the regime in which the ACE2 level enhances its propagation.

This analysis agrees with the apparently surprising data reported in Figure 3A of Ref. (Hofmann et al., 2005), which shows that ACE2 overexpression in 293T cells enhances NL63 infection three times more than SARS-CoV infection, indicating that higher receptor levels accelerate the propagation of NL63 more than that of SARS-CoV despite the latter has higher binding affinity.

## 3 Discussion

Since ACE2 is the SARS-CoV-2 receptor, we may expect that raising its level enhances the rate at which the virus propagates in the organism and worsens the outcome of the infection. However, a mathematical model of viral progression presents a regime in which the increase of the receptor level slows down the virus propagation in the organism. The observed relation between SARS-CoV-2 lethality and ACE2 levels suggests that this may be the relevant regime of SARS-CoV-2 infections, as further supported by the prediction of the age- and sex-dependent lethality of 2003 SARS-CoV.

### 3.1 Human ACE2

An important limitation of the present work is that it uses data of ACE2 protein levels in adult rat lungs (Xie et al., 2006) since similar data are not available for humans. Rat data are also consistent with observations in adult mice, which show decrease of ACE2 mRNA (Booeshaghi and Pachter, 2020) and membrane-bound ACE2 protein at old age and a general strengthening of the inflammatory arm of the RAS (Yoon et al., 2016).

Both for rodents and for humans ACE2 mRNA starts to be expressed in late foetal life (Muus et al., 2020), it is less expressed in young children than in adults (Bunyavanich et al., 2020; Saheb Sharif-Askari et al., 2020), as also found for ACE2 protein in serum (Pavel et al., 2021), it reaches a maximum at young age and then it decreases (Chen et al., 2020), see also Figure 3 of (Muus et al., 2020). In humans, membrane-bound ACE2, which is the relevant quantity for the present analysis, also decreases with age (Zhang et al., 2021) and it was found to be more abundant in children than in adult lungs (Ortiz et al., 2020), although this comparison may be debatable since it depends on the age examined and on some arbitrary thresholds.

ACE2 is removed from the cellular membrane and shed to the serum by the metalloprotease ADAM17 (Lambert et al., 2005; Xu et al., 2017) that is upregulated with age (Dou et al., 2017; Liu et al., 2019), consistent with the notion that ADAM17 is upregulated by the binding of Ang1-8 to the angiotensin II type 1 receptor (ATR1) which tend to increase with age (Yoon et al., 2016). The increase of ACE2 protein shedding with age implies that the age at which ACE2 protein expression is maximum is lower, and the rate at which it decreases with age is higher than ACE2 at the mRNA level. Since children express more than adults the receptor ATR2 that competes with ATR1 and counteracts its action (Kaschina et al., 2017), they are expected to present lower activation of ADAM17, which may partly explain why they suffer less severe Covid-19 despite having low level of ACE2. Note that ACE2 level of children does not prevent them from suffering of NL63 infections despite this virus is less efficient at binding ACE2.

Regarding sex differences, Ref. (Chen et al., 2020) found that ACE2 mRNA is lower in males than females, as in rats. Ref. (Muus et al., 2020). reached the opposite conclusion, but this seems an artefact of the fact that smoking enhances ACE2 expression (Saheb Sharif-Askari et al., 2020) and in their samples 50% of men were smokers compared to 25% of women. It has to be noted that the ACE2 gene is contained in the X chromosome, of which females have two copies. Although one of these copies is epigenetically silenced, about 15% of the X-linked genes escape this inactivation (Carrel and Willard, 2005) and heterochromatin is known to dysregulate with age. It is interesting that old female rats present almost exactly double ACE2 than males (Xie et al., 2006), as one would expect if the epigenetic silencing fades at old age. Consistently, some of the sex differences in human cardio-vascular diseases have been attributed to sex differences in the expression of ACE2, which acts as protective factor (Gupte et al., 2012).

The negative relation between ACE2 levels and severity of Covid-19 is supported by other risk and protective factors corrected for age, sex and other comorbidities in a large study in the United Kingdom (Williamson et al., 2020). Namely, being a current smoker constitute a curious protective factor (adjusted hazard ratio (AHR): 0.82−0.97), and smoking enhances ACE2 expression (Saheb Sharif-Askari et al., 2020). Contrary to single-factor analysis, diagnosed hypertension is a protective factor (AHR 0.85−0.93), which may be correlated with the fact that anti-hypertensive drugs enhance ACE2 expression (Ferrario et al., 2005). Diabetes is a risk factor (AHR 1.72−2.09), and it has been associated with reduced ACE2 expression (Batlle et al., 2010). Cardiovascular diseases and reduced kidney function are additional risk factors that are related with reduced ACE2 levels (Paz Ocaranza et al., 2020). Finally, Vitamin D deficiency is a risk factor for COVID-19 (Biesalski, 2020) that is also related with low levels of ACE2 because Vitamin D inhibits the expression of Renin, which in turn produces Ang1-10, the substrate from which is cleaved Ang1-8, which downregulates ACE2 (Ajabshir et al., 2014). Therefore, low levels of Vitamin D are expected to reduce ACE2. Vitamin D levels are decreased in ethnic groups with dark skin pigmentation living at temperate latitudes, probably due to high screening of solar radiation, providing a possible causal relationship between ethnic status and Covid-19 (AHR 1.30−1.69 for Black people, discounting socio-economic factors), once again through ACE2. Therefore, other hazard factors and protecting factors besides age and sex also support a negative correlation between ACE2 and Covid-19 lethality.

Finally, the GTEx database shows that, despite lungs are the organ that is more severely damaged by COVID-19, they do not present high expression of ACE2 mRNA (Gene Page, 2021), which is higher in tissues from reproductive organs, intestine, adipose tissue, kidney, hearth, thyroid, esophagus, breast, salivary glands and pancreas, among others. Some of these organs may be infected but they experience less damage, consistent with the negative correlation between ACE2 levels in lungs and lethality.

### 3.2 Role of ACE2 for Virus Propagation and Spike Mutant D614G

The above evidence strongly supports the negative correlation between ACE2 protein levels and severity of CoViD-19. This in turn supports the mathematical model presented here, based on the hypothesis that increased ACE2 may slow down viral propagation (Ortega-Cejas et al., 2004), which fits the CFR from Spain, Italy and Germany with $r^{2}=0.93$, 0.86 and 0.83, respectively, using two free parameters. The same hypothesis predicts the lethality profile of the 2003 SARS virus across age and sex yielding $r^{2}=0.92$, 0.87 and 0.99 using the fit parameters from Spain, Italy and Germany, respectively, and a single free parameter. This extrapolation from SARS-CoV-2 to SARS uses the ratio between the binding rate constants of the spike proteins of the two viral species, bridging the molecular and the epidemiological level.

Our mathematical model predicts that SARS has higher relative mortality for young age with respect to old age (the observed ratio is 22% for SARS compared with 1.3% for SARS-CoV-2) due to the smaller binding affinity of the SARS spike for ACE2. It also predicts that mutations that decrease the binding of SARS-CoV-2 spike may generate a strain more severe for younger age.

The spike mutant D614G, which rapidly rose to almost fixation world-wide (Korber et al., 2020), presents an opportunity to assess this prediction since it presents lower affinity for ACE2 (Yurkovetskiy et al., 2020). It propagates faster in cell cultures than the original spike (Yurkovetskiy et al., 2020) and its detected cases tend to be younger (Wagner et al., 2020), in line with the above prediction. Nevertheless, the improved propagation of D614G was attribute to the higher population of the binding-competent open configuration (Yurkovetskiy et al., 2020), thus other possible interpretations exist. A direct relation between D614G and disease severity could not be proven, but there might be an indirect one since D641G is associated with increased viral load and viral load is associated with hospitalization (Wagner et al., 2020). This interesting mutant may deserve further study both computationally, testing whether it affects the age-mortality profile in countries where detailed data are available, and experimentally, comparing its kinetics with respect to the original virus as a function of ACE2 expression.

### 3.3 Implications of the Model

An important contribution of this work is that it contradicts the idea that the increase of ACE2 should always favour virus propagation and increase the risk. This idea had important practical consequences since it lead to propose that Angiotensin receptor blockers (ARB) and ACE inhibitors (ACE-I) commonly used to treat hypertension may favour viral propagation since they upregulate the viral receptor ACE2 (Ferrario et al., 2005), and should be discontinued (Diaz, 2020; Fang et al., 2020). Medical societies firmly opposed this suggestion due to lack of evidence (ACC, 2020; EMA, 2020; ESC, 2020), and it was proposed that withdrawal of anti-hypertensive drugs in patients that need them may be harmful (Vaduganathan et al., 2020).

SARS and probably also SARS-CoV-2 degrade ACE2 (Kuba et al., 2005), with detrimental effects on the lungs on which ACE2 has a protective effect (Imai et al., 2005; Nicholls and Peiris, 2005; Imai et al., 2008; Jia, 2016). Several papers proposed that the downregulation of ACE2 is a key factor for the severity of CoViD-19 and suggested that ACE-I and ARB that limit the effects of Ang1-8 may be beneficial for CoViD-19 patients (Annweiler et al., 2020; Ciaglia et al., 2020; Gurwitz, 2020; Offringa et al., 2020; Sun et al., 2020; Verdecchia et al., 2020). A similar idea was already proposed at the time of SARS, and a retrospective meta-analysis found that the use of ARB and ACE-I provides a consistent reduction in risk of pneumonia compared with controls (Caldeira et al., 2012). In the context of CoViD-19, a meta-analysis of several studies found that ARB and ACE-I mitigate the severity of COVID-19 for patients that already take them against hypertension (Guo et al., 2020).

The negative correlation between ACE2 levels and lethality of SARS-Cov-2 described here, and the mathematical prediction that the receptor level may slow down viral progression, contradict the fear that ARB and ACE-I may benefit the virus and suggests two complementary protective roles of high ACE2 levels. On one hand, they may slow down the propagation of the virus, an effect conceptually similar to that observed in recent experiments with soluble human ACE2 (Monteil et al., 2020). On the other hand, they reduce the accumulation of Ang1-8, whose proinflammatory and prothrombotic effects are thought to underlie the most severe complications of CoViD-19. This work thus supports the idea that ARB and ACE-I used to treat high blood pressure may limit the most adverse manifestations of CoViD-19.

A note of caution is required for the effect of these drugs on the bradykinin system. This system is strongly coupled with the RAS and causes vasodilation, reduces blood pressure and increases vascular permeability. Its over-activity can lead to increased inflammation, thrombosis and angioedema in the lungs, and it was proposed that it also mediates the severe manifestations of COVID-19 (Garvin et al., 2020; Nicolau et al., 2020; van de Veerdonk et al., 2020). The bradykinin system consists of two axes. The first one is downregulated by ACE2, which degrades the signalling peptide des-Arg^9^-bradykinin (DABK) whose receptor BK1R is upregulated by Ang1-8 (in turn downregulated by ACE2) bound to the receptor ATR1. Stimulation of this axis may lead to release of pro-inflammatory chemokines, lung inflammation and injury (Sodhi et al., 2018) and is expected to be reduced through ARB and ACE-I, which would exert a protective role. The other axis is downregulated by ACE, which degrades the signalling peptide BK, whose receptor BK2R is in turn activated by Ang1-7 and Ang1-9 produced by ACE2, and is stimulated by Ang1-8 bound to the receptor ATR2 (Kurisu et al., 2003). Thus, ACE2, ATR1 receptor blockers and even more ACE-I can upregulate the BK2R axis with pathological consequences, as observed in severe side-effects of ACE-I (Wood, 1995), and their use should be limited in the presence of hypotension. Nevertheless, studies of the effect of these drugs on COVID-19 patients found an overall positive balance.

Of course, clinical trials are necessary to establish whether ARB and ACE-I have a positive, negative or neutral effect on CoViD-19 severity. To this aim, the clinical trials NCT04312009 and NCT04311177 started in April 2020 at the University of Minnesota.

### 3.4 Positive Feedback Loop

It is noteworthy that degradation of ACE2 increases the level of Ang1-8, which in turn binds the ATR1 receptor and down-regulates ACE2 even further both at the mRNA and at the protein level (Deshotels et al., 2014). Thus, the SARS-CoV-2 infection may trigger a dangerous positive feedback loop that strongly raises Ang1-8, exacerbating inflammatory response (Agarwal et al., 2013; Satou et al., 2018) and coagulation problems (Lip, 2000; Remková and Remko, 2010), frequent complications of severe CoViD-19 patients (Diamond, 2020; Tseng et al., 2020). Positive correlation between Ang1-8 levels and viral load has been reported in CoViD-19 patients (Liu et al., 2020), supporting the link between severe CoViD-19 and dysregulation of the RAS.

Under this point of view, ARBs appear to be more favourable than ACE-I because they can interfere with the positive feedback loop of Ang1-8 and because Ang1-8 can be generated by other proteases if ACE is inhibited (Paz Ocaranza et al., 2020).

### 3.5 RAS Proteins as Prognostic Factors

The results presented here suggest a prognostic role for the measurements of key components of the RAS and the bradykinin system in bronchial aspirated lavage samples and in the serum, which may predict the severity of the disease already at an early stage and may allow detecting risk groups that need higher protection besides the elder, as supported by the association between ACE2 and known risk and protecting factors against CoViD-19 (Williamson et al., 2020). We are currently investigating this possibility through retrospective studies.

## 4 Methods

### 4.1 Case-Fatality-Rates and Expression Data

Case fatality rates (CFR) were taken from public sources (Istituto Superiore di Sanità, 2020; OPS, 2020; Robert Koch Institute, 2020) for CoViD-19 in Italy, Spain and Germany, respectively, and from Ref. (Karlberg et al., 2004). for the 2003 SARS outbreak in Hong-Kong. At the beginning of an outbreak, CFR underestimate the true fatality rate because their calculation assumes that all people currently infected will recover, which unfortunately is not true. This effect may not be uniform across age-sex classes if patients of some classes tend to die more rapidly, as assumed by the model. However, at a late epidemic stage this effect is expected to be small. On the other hand, CFR overestimate the true fatality rate because of undetected cases that tend to lower the denominator. Since age-sex classes with higher lethality also tend to have more severe cases and less undetected cases, the overestimation is larger for classes with smaller lethality, with the consequence of reducing the differences among classes for larger fraction of undetected cases. The data that we used do not allow correcting for this bias, which may account for some of the differences in the fit parameters.

Expression data presented in Ref. (Xie et al., 2006). were grouped in three age classes of 3 (young), 12 (middle) and 24 months (old). CFR were presented in bins of 10 years, and I grouped them in three equally spaced groups 0–29 (young), 30–59 (middle) and ≥60 years (old). Grouping the 20–29 years class with the middle age gave similar results with approximately exponential decrease of lethality with ACE2 expression.

For SARS CFR (Karlberg et al., 2004), ages were grouped differently: 0–44 (young), 45–74 (middle) and ≥75 (old). To compare these groups with those of SARS-CoV-2, I interpolated expression data of ACE2 *A* for these groups as A(0−44)=0.667A(0−29)+0.333A(30−59), A(45−74)=0.5A(30−59)+0.5A(≥60) and A(≥75)=0.667A(≥60). Other schemes gave qualitatively similar results: The CFR decreases approximately exponentially with *A* and the exponent is smaller than for SARS-CoV-2.

### 4.2 Mathematical Model of Viral Propagation

The simplest mathematical model of viral propagation in an organism considers three populations: uninfected cells U(t), free virus V(t) that enter the cells with rate $k_{1}U\left(t\right)V\left(t\right)$ (adsorption) and are cleared with rate *c*, and infected cells I(t) that produce new virus at rate $k_{2}YI\left(t\right)$ (*Y* is the number of viable virus produced by an infected cell) after a delay time τ until they ultimately die. The viral population cannot grow if the rate of virus production is lower than the clearance rate, which gives a minimum adsorption rate $k_{1}$ (Smith and Perelson, 2011). Here I express the adsorption rate as a function of the receptor density *A*, $k_{1}=kA$, which translates into the minimum receptor density $A^{\text{min}}=c/\left(kU_{0}Y\right)$ ($U_{0}$ is the initial concentration of susceptible cells). A spatially explicit version of this model in which virus diffuse through susceptible cells (Fort and Méndez, 2002) was solved analytically to explicitly represent the viral velocity *v* as a function of the model parameters (Ortega-Cejas et al., 2004). Here I describe this solution in terms of the receptor density. The authors describe two regimes (see Eqs 15, 16 of Ref. (Ortega-Cejas et al., 2004). with the notation $k_{1}=kA$, $f\propto U_{0}$ and $D_{\text{eff}}\equiv D\left(U_{\text{max}}-U_{0}\right)/\left(U_{\text{max}}+U_{0}/x\right)=D_{e}$, where $D_{e}$ is the effective diffusion constant that depends on cell shape and on $U_{0}$): 1) For small *A* ($A<1/\left(kU_{0}\tau Y\right)$ and $A<1/\left(kU_{0}k_{2}\right)$), the viral velocity increases with *A*, but less then linearly, as $v=2\sqrt{D_{e}\frac{kAU_{0}Y}{1+kAU_{0}Y}}$. 2) For intermediate receptor density $1/\left(kU_{0}\tau Y\right)<A<1/\left(kU_{0}k_{2}\right)$ the viral velocity is given by $v=\sqrt{2D_{e}/\tau }$, and it is almost independent of *A* (Ortega-Cejas et al., 2004) so that the viral progression is not enhanced by the expression of the receptor. 3) The formulas presented in the paper are also valid in the third regime of very high receptor density, when kA is larger than the rate at which viral particles are produced: $A>1/\left(kU_{0}\tau Y\right)$ and $A>1/\left(kU_{0}k_{2}\right)$. This regime was not explicitly discussed in Ref. (Ortega-Cejas et al., 2004), but it can be easily computed in the limit of large adsorption in which the parameter $\kappa =\left(kAU_{0}\right)/k_{2}$ defined on page 2 of Ref. (Ortega-Cejas et al., 2004). is large. Keeping only the dominant terms in Eq. 9 on the same page, we obtain $a_{0}\approx b_{0}\kappa$, $a_{1}\approx b_{1}\kappa^{2}$, $a_{2}\approx b_{2}\kappa^{3}$, $a_{3}\approx b_{3}\kappa^{3}$. Substituting these expressions in Eq. 8, which is the equation for the wave velocity, and dividing by κ, we obtain the equation $b_{0}+b_{1}\left(\kappa \xi \right)+b_{2}{\left(\kappa \xi \right)}^{2}+\left(b_{3}/\kappa \right)\text{*}{\left(\kappa \xi \right)}^{3}=0$, which indicates that, in the limit of fast adsorption, κξ tends to a constant. Since $\xi =v^{2}/\left(D_{e}k_{2}\right)$, this scaling can be written in the form $v\propto \sqrt{D_{e}k_{2}/\kappa }$. Therefore, counter-intuitively, in this regime the viral progression slows down with receptor density as $v\propto k_{2}\sqrt{D_{e}/kAU_{0}}$.

The time that it takes for the virus to propagate through the upper respiratory tract (URT) can be estimated as $t_{U}=l_{U}/v$. The lungs are a classical example of a fractal organ with fractal dimension $d_{F}\approx 2.35$ (Weibel, 1991). For the ease of the calculation, I approximate the diffusion velocity *v* on the lungs with the diffusion velocity for an one-dimensional system given in Ref. (Ortega-Cejas et al., 2004), although this is not completely correct. In this approximation, the number of infected lung cells grows with time as $I\left(t\right)\propto {\left(vt\right)}^{d_{F}}$. As cells get infected, the receptor density in the lungs decreases as $A\left(t\right)=A\left(0\right)\left(1-I\left(t\right)/l_{L}^{d_{F}}\right)$ and it reaches the critical level $A_{c}$ after the time $t_{L}=(l_{L}/v({\left(1-A_{c}/A_{0}\right)}^{1/d_{F}}\approx =\frac{l_{L}}{v}(1-\frac{A_{c}}{d_{F}}\frac{1}{A\left(0\right)})$. I used the approximation $A_{c}\ll A\left(0\right)$, and A(0) is the receptor density at the beginning of the infection, which in the main text is simply denoted as *A*. Summing these two times, I estimate the time at which death occurs as $t_{d}\approx \left(1/v\right)\left[l_{U}+l_{L}\left(1-\frac{A_{c}}{d_{F}}\frac{1}{A}\right)\right]$.

I consider three situations: 1) *v* is independent of *A*; 2) *v* decreases as $1/\sqrt{kA}$ and almost all the cells in the lungs must be infected to produce the death, i.e., $A_{c}=0$; 3) *v* decreases with *A* and $A_{c}>0$. Although case 2) is a special case of 3) with $A_{c}=0$, it is convenient to treat it separately for the sake of comparison, because case 1) and 2) have the same number of free parameters and their fits can be compared in equal conditions to determine which proposed mechanism, the protective effect of ACE2 1) or the decrease of viral velocity 2) can better explain the observed negative correlation between ACE2 and lethality.

In each situation, the death time $t_{d}$ depends on *A* as $t_{d}\propto 1-\frac{A_{c}}{d_{F}}\frac{1}{A}$ (Model 1), $t_{d}\propto \sqrt{k}\sqrt{A}$ (Model 2), $t_{d}\propto \sqrt{k}\left(\sqrt{A}-\frac{A_{c}}{d_{F}}\frac{1}{\sqrt{A}}\right)$ (Model 3).

In the model, death occurs if $t_{d}$ is smaller than the time $t_{i}$ needed by the immune system to control the virus, which is modelled either as an exponential (E) or a Gaussian (G) random variable. In the first case, the probability that $t_{i}$ is larger than $t_{d}$ can be computed as $P_{d}=\text{exp}\left(-t_{d}/T\right)$, where *T* is the average value of $t_{i}$. In the Gaussian case, the probability is well approximated as $P_{d}=C\text{exp}\left(-{\left(t_{d}-\mu \right)}^{2}/\left(2\sigma^{2}\right)\right)$. I combine these expressions with the three models of $t_{d}$ versus *A*, I approximate the Gaussian integral with its saddle point to obtain an analytic expression with few free parameters and adopt the hypothesis that $\mu <t_{d}$, which is justified by empirical observations (the development of antibodies takes few days while death tends to occur after two or three weeks, and robust antibody response was observed even in severe Covid-19 patients (Pierce et al., 2020). Finally, I group together terms with the same power of *A* and obtain six mathematical models of the lethality $P_{d}$ as a function of the initial level of ACE2 *A*:

$$-\text{ln}\left(P_{d}\right)\approx \{\begin{array}{cc} -\frac{a}{A}+b\;\;\left(1\text{E}\right) & \frac{a}{A^{2}}-\frac{b}{A}+c\;\;\left(1\text{G}\right)\\ a\sqrt{A}+b\;\;\left(2\text{E}\right) & aA-b\sqrt{A}+c\;\;\left(2\text{G}\right)\\ a\sqrt{A}-\frac{b}{\sqrt{A}}+c\;\;\left(3\text{E}\right) & aA-b\sqrt{A}+\frac{c}{\sqrt{A}}+d\;\;\left(3\text{G}\right) \end{array}$$

*a*, *b* and *c* are positive fitting parameters. In Eq. 3G, there are five terms proportional to *A*, $\sqrt{A}$, $1/\sqrt{A}$, 1/A and constant, corresponding to five fitting parameters. In order to reduce the free parameters, I neglected the term proportional to 1/A, obtaining Eq. 3G.

### 4.3 Fit of the Models

The fitting parameters *a* and *b* are determined through regularized fits performed with rescaled ridge regression (Bastolla and Dehouck, 2019), which minimizes the quadratic error plus the term $\text{Λ}\left(a^{2}+b^{2}\right)$ that penalizes large values of the parameters. The regularization is necessary to avoid amplifying the noise due to covariant explanatory variables, as in the present case, and it allows more robust parameter estimation, often avoiding that they acquire unphysical values with incorrect sign, at the price of some bias. Rescaled ridge regression yields non-vanishing parameters even in the limit of large Λ, overcoming a drawback of other regularization schemes, and it fixes the parameter Λ based on an analogy with statistical mechanics at the transition between the phase dominated by the noise and the one dominated by the bias. For ridge regression there is no analytic formula to determine the statistical error of the fitting parameters, therefore I applied a bootstrap approach, repeating the fit while eliminating each of the data points and computing the standard deviation of the fitting parameters numerically.

Model (2G) coupled to the Gaussian distribution depends on three parameters but only the parameters *a* and *b* were fitted while *c* was fixed, fitting $-\text{ln}\left(P_{d}\right)-c=aA-b\sqrt{A}$. The parameter *c* was determined not by minimizing the fit error but by selecting the value of *c* that yields 50% relative error on the parameters *a* and *b*. The plots shown in Figure 1 were obtained in this way.

### 4.4 Prediction for SARS

The models fitted to SARS-CoV-2 were rescaled in order to apply them to SARS-CoV, adopting the ratio between the binding rates of the spike proteins of both viruses to ACE2. The most precise measures available in the literature are $k_{\text{SARS}-2}=\left(2.3\pm 1.4\right)10^{5}nM^{-1}s^{-1}$ and $k_{\text{SARS}}=\left(1.7\pm 0.7\right)10^{5}nM^{-1}s^{-1}$ (table 1 in (Walls et al., 2020)). Although the error bars are huge, the greater rate constant of SARS-CoV-2 agrees with the more precisely determined binding affinity from the same table ($K_{\text{SARS}-2}=\left(1.2\pm 0.1\right)nM$ and $K_{\text{SARS}}=\left(5.0\pm 0.1\right)nM$), and from Ref. (Wrapp et al., 2020) that indicates that the spike protein of SARS-CoV-2 has greater affinity for ACE than the one of SARS-CoV. In that paper only one experiment was performed instead of five in Ref. (Walls et al., 2020). and the binding rate constant was greater for SARS-CoV, which is consistent with the large statistical errors measured in Ref. (Walls et al., 2020). Thus, although the available data is quite noisy, the best available evidence suggests that the binding rate of SARS-CoV-2 spike protein is on the average faster than for SARS and the binding is more stable, which may also contribute to faster adsorption giving the virus more time to perform membrane fusion.

For predicting the CFR of the 2003 SARS outbreak, I used the parameters of SARS-CoV-2 and rescaled them with the ratio between the kinetic binding constant $k^{\text{on}}$ of the two spike proteins: $a_{\text{SARS}}=a_{\text{SARS}-2}/1.35$ and $b_{\text{SARS}}=b_{\text{SARS}-2}/\sqrt{1.35}$. I obtained the lethality profile as $\text{exp}\left(-a_{\text{SARS}}A+b_{\text{SARS}}\sqrt{A}\right)$, where *A* is the ACE2 level of each sex and age class, and multiplied it times a constant factor that accounts for the different fraction of undetected cases, which was the only free parameter determined through a fit.

## Data Availability

Publicly available datasets were analyzed in this study. This data can be found here: https://www.epicentro.iss.it/coronavirus/bollettino/Bollettino-sorveglianza-integrata-COVID-19_2-aprile-2020.pdf https://www.mscbs.gob.es/profesionales/saludPublica/ccayes/alertasActual/nCov/documentos/Actualizacion_76_COVID-19.pdf https://www.rki.de/DE/Content/InfAZ/N/Neuartiges_Coronavirus/Situationsberichte/2020-04-28-en.pdf.
