# Supplementary material for: Mathematical Model of SARS-Cov-2 Propagation Versus ACE2 Fits COVID-19 Lethality Across Age and Sex and Predicts That of SARS
Source: Front Mol Biosci. 2021 Jul 12;8:706122. doi: 10.3389/fmolb.2021.706122 (PMC8311794; doi:10.3389/fmolb.2021.706122)
Supplement: Supplementary file 1 [file DataSheet1.PDF]

Supplementary Material for the paper Mathematical model of SARS-Cov-2 propagation versus ACE2 fits COVID-19 lethality across age and sex and predicts that of SARS

Ugo Bastolla[(1)]

[(1)] Centro de Biologia Molecular "Severo Ochoa"

CSIC-UAM Cantoblanco, 28049 Madrid, Spain. email: ubastolla@cbm.csic.es

Fit[a]	$v(A)$[b]	$A^c_{\text{cr.}}$	$P(t_{\text{i}})$[d]	$-\ln(P_d)$[e]	Error[f]	a[g]	b[h]	c[i]	Data[j]
					0.03	5.2 ± 0.1	1.1 ± 0.1		ES
exp				$aA + b$	0.09	6.4 ± 0.3	0.5 ± 0.2		IT
					0.11	6.3 ± 0.4	1.6 ± 0.2		DE
					0.37	0.5 ± 0.2	5.5 ± 0.4		ES
1E	Const	> 0	E	$-\frac{a}{A} + b$	0.41	0.6 ± 0.3	6.0 ± 0.6		IT
					0.53	0.5 ± 0.3	6.6 ± 0.7		DE
					0.07	6.7 ± 0.5	-0.8 ± 0.4		ES
2E	Decr	0	E	$a\sqrt{A} + b$	0.14	8.2 ± 0.8	-1.7 ± 0.6		IT
					0.17	7.8 ± 1.0	-0.5 ± 0.7		DE
					0.13	4.5 ± 1.5	0.4 ± 0.2	1.6 ± 1.0	ES
3E	Decr	> 0	E	$a\sqrt{A} - \frac{b}{\sqrt{A}} + c$	0.20	5.2 ± 0.8	0.7 ± 0.4	1.6 ± 0.5	IT
					0.22	5.5 ± 1.1	0.4 ± 0.4	1.9 ± 0.7	DE
					0.04	0.24 ± 0.1	2.5 ± 0.5	8.1 ± 0.6	ES
1G	Const	> 0	G	$\frac{a}{A^2} - \frac{b}{A} + c$	0.10	0.34 ± 0.3	3.3 ± 1.8	9.4 ± 2	IT
					0.14	0.34 ± 0.4	3.3 ± 2	10 ± 2	DE
					0.01	6.6 ± 1	0.7 ± 1.4	0.7 ± 0.4	ES
2G	Decr	0	G	$aA - b\sqrt{A} + c$	0.04	13 ± 1.5	8 ± 2	2.4 ± 0.9	IT
					0.06	16 ± 1	13 ± 2	5.2 ± 0.5	DE
					0.01	7.3 ± 0.1	0.9 ± 0.1	0.3 ± 0.1	ES
3G	Decr	> 0	G	$aA - b\sqrt{A} + \frac{c}{\sqrt{A}}$	0.04	11 ± 0.8	4.2 ± 0.9	0.4 ± 0.2	IT
					0.06	12 ± 0.8	5.2 ± 1	1.1 ± 0.1	DE
					0.01	6.9 ± 0.5	1.1 ± 0.5	0.85	ES
2G2	Decr	0	G	$aA - b\sqrt{A} + c$	0.07	7.6 ± 0.5	1.1 ± 0.4	0.525	IT
					0.09	8.7 ± 1	2.0 ± 1	1.425	DE
					0.08	5.09	0.97	0.01	SA
2G1	Decr	0	G	$aA - b\sqrt{A} + c$	0.13	5.66	0.93	0.10	SA
					0.01	6.49	1.74	0.58	SA

Table 1: **Fit results.** (a) Type of fit. (b) Dependence of viral velocity on receptor level A. I test only the constant and decreasing regime because the increasing regime contradicts the data. (c) Critical level of ACE2 below which the patient dies. If $A_{\text{crit.}} = 0$ death happens later and the CFR depends less on the initial level of ACE2. (d) Distribution of the response time of the immune system, either exponential (E) or Gaussian (G). (e) Functional form of the logarithm of the death probability (estimated as CFR) versus the initial receptor level A computed with the mathematical model under hypothesis b, c and d. (f) Relative mean square error $1-r^2$ of the fit regularized with rescaled ridge regression (i.e. the error is not the minimal possible), imposing that all fit parameters are positive. (g-i) Fit parameters and statistical error computed with bootstrapping. (j) Fitted CFR data. ES=Spain, IT=Italy, DE=Germany, SA=SARS 2003 Hong Kong. In fit 2G2 only the parameters a and b are fitted while c is set to get a given value of the relative error of the parameters. In fit 2G1, for the 2003 SARS, only the multiplicative parameter c is fitted while a and b are predicted based on the corresponding parameters of Spain, Italy and Germany and the ratio between the kinetic rate constant of the spike proteins of SARS-Cov and SARS-Cov-2. Note that two-parameter fits from Italy and Germany coincide within the error except for a multiplicative factor (additive in the logarithm) despite the fraction of undetected cases of the two countries are very different.
